# Partial p53 reactivation is sufficient to induce cancer regression

**DOI:** 10.1186/s13046-022-02269-6

**Published:** 2022-03-02

**Authors:** Boris Klimovich, Laura Meyer, Nastasja Merle, Michelle Neumann, Alexander M. König, Nikolaos Ananikidis, Corinna U. Keber, Sabrina Elmshäuser, Oleg Timofeev, Thorsten Stiewe

**Affiliations:** 1 Institute of Molecular Oncology, Universities of Giessen and Marburg Lung Center (UGMLC), Philipps-University, Marburg, Germany; 2 Clinic of Diagnostic and Interventional Radiology, Core Facility 7T-small animal MRI, Philipps-University, Marburg, Germany; 3 Institute for Pathology, University Hospital Marburg, Philipps-University, Marburg, Germany; 4 German Center for Lung Research (DZL), Philipps-University, Marburg, Germany; 5 Genomics Core Facility, Philipps-University, Marburg, Germany

**Keywords:** p53, Tumor suppressor gene, p53 reactivation, Molecular therapy, Leukemia, Lymphoma, Mouse models

## Abstract

**Background:**

Impaired p53 function is one of the central molecular features of a tumor cell and even a partial reduction in p53 activity can increase the cancer risk in mice and men. From a therapeutic perspective it is noteworthy that tumor cells often become addicted to the absence of p53 providing a rationale for developing p53 reactivating compounds to treat cancer patients. Unfortunately, many of the compounds that are currently undergoing preclinical and clinical testing fail to fully reactivate mutant p53 proteins, raising the crucial question: how much p53 activity is needed to elicit a therapeutic effect?

**Methods:**

We have genetically modelled partial p53 reactivation using knock-in mice with inducible expression of the p53 variant E177R. This variant has a reduced ability to bind and transactivate target genes and consequently causes moderate cancer susceptibility. We have generated different syngeneically transplanted and autochthonous mouse models of p53-deficient acute myeloid leukemia and B or T cell lymphoma. After cancer manifestation we have activated E177R expression and analyzed the in vivo therapy response by bioluminescence or magnetic resonance imaging. The molecular response was further characterized in vitro by assays for gene expression, proliferation, senescence, differentiation, apoptosis and clonogenic growth.

**Results:**

We report the conceptually intriguing observation that the p53 variant E177R, which promotes de novo leukemia and lymphoma formation, inhibits proliferation and viability, induces immune cell infiltration and triggers cancer regression in vivo when introduced into p53-deficient leukemia and lymphomas. p53-deficient cancer cells proved to be so addicted to the absence of p53 that even the low-level activity of E177R is detrimental to cancer growth.

**Conclusions:**

The observation that a partial loss-of-function p53 variant promotes tumorigenesis in one setting and induces regression in another, underlines the highly context-specific effects of individual p53 mutants. It further highlights the exquisite sensitivity of cancer cells to even small changes in p53 activity and reveals that changes in activity level are more important than the absolute level. As such, the study encourages ongoing research efforts into mutant p53 reactivating drugs by providing genetic proof-of-principle evidence that incomplete p53 reactivation may suffice to elicit a therapeutic response.

**Supplementary Information:**

The online version contains supplementary material available at 10.1186/s13046-022-02269-6.

## Background

The tumor suppressor gene *TP53* encodes the p53 transcription factor, which regulates target genes in multiple pathways to counteract the expansion of malignant cells [1–3]. p53 thereby poses a major barrier to tumor development, explaining why tumorigenesis strongly selects for cells with inactivated p53 [3]. Loss of p53 function (LOF) can result from p53 degradation or sequestration by viral oncoproteins or cellular inhibitors like Mdm2 or Mdmx [4, 5]. In approximately 50% of all tumors, LOF is caused by somatically acquired genetic alterations affecting the *TP53* gene directly [5]. This includes large deletions encompassing the entire gene as well as small non-sense, frameshift or missense mutations, which can differ massively in their functional impact [5, 6]. Different from other tumor suppressor genes, most *TP53* mutations are missense mutations, explained in part by dominant-negative and tumor-promoting neomorphic (gain-of-function, GOF) properties of at least some of the mutant proteins [7–10]. Of note, full p53 activity is essential for optimal tumor suppression as even a partial loss of p53 function increases the cancer risk in mice and causes hereditary cancer susceptibility in humans [11–17].

Importantly, restoration of p53 in p53-deficient tumor cells was found to be detrimental, usually resulting in cell death or loss of proliferative capacity due to senescence or differentiation [18]. For example, in p53ER^TAM^ mice with tamoxifen-switchable p53 activity, EμMyc-driven Burkitt-like lymphomas develop in the p53-off state, but undergo rapid regression when p53 is switched on [19]. This was observed even when p53 inactivation was not the tumor-initiating driver lesion and acquired only later during tumor progression [20]. Together with other studies in independent mouse cancer models, this firmly established that tumor cells can become addicted to the loss of p53 [21–24]. Moreover, these studies demonstrated the therapeutic potential of p53 reactivation and provided critical support for the development of p53-reactivating drugs as cancer therapeutics [25, 26].

Meanwhile a growing number of p53 reactivating compounds has been developed. While Mdm2 and Mdmx inhibitors are being evaluated for treatment of tumors with wild-type p53, diverse strategies have been proposed to target tumors with *TP53* mutations [25, 26]. As straightforward as reactivation may seem, as challenging it turns out in practice. The most common *TP53* mutations either destroy DNA contact residues or destabilize the thermodynamically labile p53 DNA binding domain and cause its denaturation at normal body temperature [27]. The most direct reactivation approaches aim to refold the pool of mutant p53 proteins, that have accumulated in the tumor cells, into a native conformation [28, 29]. However, as more than 2000 different mutant proteins have been identified in cancer patients, a universal reactivation strategy is unrealistic. As such, many reactivation compounds target only single or groups of mutants [30]. For example, PhiKan083 specifically targets a surface crevice created by the Y220C mutation [31]. Metallochaperones increase the intracellular availability of zinc to rescue folding of zinc-binding site mutants like R175H [32]. Arsenic trioxide (ATO) binds to a cryptic allosteric site formed by Arsenic-coordinating cysteines and stabilizes the native fold of a subset of p53 mutants [33]. The clinically most advanced compound APR-246 (PRIMA-1^Met^, Eprenetapopt), is converted to the thiol-reactive metabolite methylene quinuclidinone (MQ) and proposed to alkylate and thereby reactivate structural mutants, but also, in a mechanistically poorly understood manner, some DNA contact mutants [34–36]. In combination with azacytidine, APR-246 showed promising therapeutic responses in phase II studies involving myelodysplastic syndrome (MDS) and acute myeloid leukemia (AML) patients [37, 38]. However, MQ also reacts with numerous other cellular cysteines, forming a thiol-bound drug reservoir, and through depletion of glutathione (GSH) and inhibition of the GSH and thioredoxin antioxidant systems exhibits cytotoxic activities [39, 40]. Other reactivating compounds also display considerable p53-independent toxicity that certainly adds to, if not determines, the therapeutic effects [30]. This raises the critical question: how much of the observed cytotoxicity is due to p53 reactivation [18]? Last but not least, the mutant p53 refolding activity of various reactivating compounds differs by at least two orders of magnitude not only between compounds but also between individual mutants [33]. Together these findings indicate that pharmacological mutant p53 reactivation is continuously improving, but is currently still far from optimal. This brings up an even more important question: to what extent does p53 have to be restored [18]? Is full reactivation required to achieve a therapeutic response or is partial reactivation already sufficient?

Here, we have modelled partial p53 reactivation using a conditional knock-in mouse with inducible expression of the partial LOF variant p53^E177R^ (short: E177R), which corresponds to human E180R. The E177R mutation disrupts an intermolecular salt-bridge with R178 (human R181) that is required for cooperative DNA binding [41–43]. The decrease in cooperativity reduces DNA binding and target gene activation [15, 44, 45]. As a result, E177R causes cancer susceptibility and cooperates with various oncogenes to promote cancer development, including lung and pancreatic adenocarcinoma and various types of leukemia and lymphoma [15, 43, 46, 47]. Here, we report that in p53-deficient leukemia and lymphoma models the effects of E177R expression are entirely opposite: E177R inhibits cell proliferation and viability and induces cancer regression. p53-deficient cancer cells prove to be so addicted to the absence of p53 that the residual activity of E177R is detrimental for cancer growth. As such, the study provides genetic proof-of-concept that incomplete p53 reactivation can make a therapeutic impact.

## Materials and methods

### Animal experiments

All mouse experiments were performed according to the German Animal Welfare Law (TierSchG) and were approved by the local authorities. Mice were housed in open cages, on a 12 h light/dark cycle, fed a standard housing/breeding diet (Altromin) and received water ad libitum. The following mouse strains were used: B6.129S/Sv-Trp53tm1Thst (*Trp53*^LSL-E177R^) [15], B6.129-Gt (ROSA)26Sortm1(cre/ERT2)Tyj/J (CreER^T2^) [22], B6.Cg-Tg (IghMyc)22Bri/J (EμMyc) [48], and B6.129-Trp53 < tm1Brd>/TacThst (p53KO) [49]. In transplantation experiments, F1 hybrids of C57BL/6 J (B6) and 129S1/SvImJ (129) or B6(Cg)-Tyrc-2 J/J (B6 albino) and 129X1/SvJ (129 albino) were used as recipients. Generation of the leukemia and lymphoma, monitoring of disease development by bioluminescence imaging (BLI) and therapy were performed as described earlier [50].

For the AML model with Cre-inducible expression of p53^E177R^, double-heterozygous *Trp53*^LSL-E177R/+^; Rosa26^CreERT2/+^ mice were intercrossed. Fetal liver cells from *Trp53*^LSL-E177R/LSL-E177R^; Rosa26^CreERT2^ embryos (E14–16) were isolated, transduced with retroviruses expressing the *AML1/ETO9a* fusion oncogene (co-expressed with GFP) and *Nras*^G12D^ oncogene (co-expressed with firefly luciferase) and 10^6^ cells were transplanted into 129 albino recipients as described [46, 50]. Recipients were lethally irradiated (7 Gy) 24 h before transplantation using the X-RAD 320iX system. Recipients were provided with neomycin-supplemented water (1.6 g ml^− 1^, pH 3) starting 2 days before transplantation until 3 weeks after. For in vivo p53 reactivation, recipient 129 albino/B6 albino F1 hybrid mice were sublethally irradiated and injected i.v. with 10^6^ AML cells. Both male and female mice were used as recipients for female AML cells with and without reactivatable p53. Leukemia progression was monitored by BLI: mice were imaged under isoflurane anesthesia 5 min after i.p. injection of 100 μl of D-luciferin solution (15 mg ml^− 1^) using an IVIS 100 imaging system (Xenogen).

For the EμMyc lymphoma model, double-heterozygous *Trp53*^LSL-E177R/+^; *Rosa26*^CreERT2/+^ females were bred with EμMyc transgenic males. Lymphomas from EμMyc; *Trp53*^LSL-E177R/^ offspring mice with or without CreER^T2^ were used for transplantation into 129/B6 F1 hybrid recipient mice as described [50]. After disease onset was confirmed by palpation of enlarged mandibular, axillary or subiliac lymph nodes, mice were treated 1 week with daily i.p. injections of 1 mg tamoxifen. Mice were euthanized when pre-defined humane endpoint criteria were reached. Control experiments for Cre-mediated toxicity were performed with EμMyc; *Rosa26*^CreERT2/+^ lymphomas. Both male and female donor and recipient mice were used.

For the spontaneous T-lymphoma model, both male and female *Trp53*^LSL-E177R/LSL-E177R^; *Rosa26*^CreERT2^ mice were examined with magnet-resonance tomography using 7 T Clinscan 70/30 USR (Bruker) as described [51]. T2 weighted sequences triggered on respiration in transverse and coronal orientation were used for anatomical imaging of the thymus. The total measurement time was approximately 28 min per mouse. Tumor size was measured using RadiAnt DICOM Viewer and tumor volume was calculated with the ellipsoid formula V = 4/3*π*abc. *Trp53*^LSL/LSL^ mice without Cre were used as a control cohort. The first imaging was performed at the age of 120 days or earlier for mice that showed clinical symptoms of thymic lymphoma (weight loss, hunchback posture, shortness of breath).

For reactivation of E177R expression via Cre-mediated recombination in vivo, mice were injected i.p. for 7 consecutive days with 100 μl of 10 mg ml^− 1^ tamoxifen (Sigma) in sterile corn oil. For mock treatment, mice were injected with 100 μl of corn oil only.

### Cell culture

For generation of mouse AML cell lines, primary tumors (spleen, bone marrow) were mechanically disrupted by mashing through 70 μm EASYstrainer (Greiner). After erythrocyte lysis (5 min at RT in ACK buffer, Thermo Fisher), cells were collected by centrifugation, resuspended and cultured in B-cell medium (DMEM:IMDM 1:1, Life Technologies), 20% fetal bovine serum (FBS, Sigma-Aldrich), 100 U ml^− 1^ penicillin/streptomycin (Life technologies), 5 × 10^− 5^ M 2-mercaptoethanol) supplemented with 0.2 ng ml^− 1^ murine IL3, 2 ng ml^− 1^ IL6 and 20 ng ml^− 1^ SCF (all from Immunotools). Cells were maintained on multi-well plates for suspension cells (Greiner) at ambient oxygen in a humidified cell culture incubator (37 °C with 5% CO_2_). For colony formation assay, 50,000 cells were plated in MethoCult™ GF M3434 medium (STEMCELL Technologies, 1.5 ml per well on 6-well plate) and colonies were counted after 7 days.

### CRISPR-Cas9

For generation of AML cells with CRISPR-mediated p53 knock-out, *Trp53*^LSL-E177R/LSL-E177R^; *Rosa26*^CreERT2^ leukemia cells were transduced with pMSCV-Cas9-Blast retrovirus. For pMSCV-Cas9-Blast plasmid, the puromycin resistance (*pac*) gene in the pMSCV-Cas9-Puro plasmid (RRID: Addgene_65655) was replaced with the blasticidin-S resistance gene (*bsr*) PCR-amplified from lentiCas9-Blast plasmid (RRID: Addgene_52962) using primer pair 5′-catgcAAGCTTccaccatggccaagcctttgtctcaag and 5′-gatcgATCGATttagccctcccacacataacc using HindIII and ClaI restriction sites, respectively. MSCV-Cas9-Blast retrovirus was packaged using Platinum-E cells as described [51] and used for spin-infection in B-cell medium supplemented with 6 μg ml^− 1^ polybrene (Sigma). AML cells were spin-infected 4 times (600×g, 40 min) in 24-well tissue culture plates (Greiner) coated with 40 μg ml^− 1^ RetroNectin (Takara). After 1 week of selection with 50 μg ml^− 1^ Blasticidin S (Invivogen), 5000 cells were plated on 35 mm tissue culture dish (Greiner) in MethoCult™ GF M3434 medium and grown for 2 weeks. Single colonies were picked, expanded and screened for Cas9 expression by Western blotting. One validated single cell clone (AML-Cas9) was used for CRISPR editing. Oligos, encoding control (scrambled) sgRNA (sense 5′-caccgaaatgtgagatcagagtaat-3′, antisense 5′-aaacattactctgatctcacatttc-3′) or sgRNA targeting *Trp53* locus [50], were cloned into lentiviral SGL40C.EFS.RFP657 vector (gift from Dirk Heckl, RRID:Addgene_69147 [52]) via BsmBI site using Golden Gate cloning. Lentiviruses were produced in HEK293-T cells and used for infection of the AML-Cas9 cell clone [20]. Infection efficiency was analyzed by flow cytometry for RFP 48 h after infection and the pool of cells was directly used for transplantation. CRISPR-mediated *Trp53* knock-out was confirmed by Sanger sequencing and InDel analysis using the TIDE algorithm [53].

### Flow cytometry

Immunophenotyping and analysis of differentiation of leukemia cells was performed as described [50] on an Accuri C6 Plus flow cytometer (BD Biosciences) with the following antibodies: mouse Gr1-PE (Milteny Biotec #130–102-426, RRID: AB_2659861), mouse CD11b-APC (Milteny Biotec #130–091-241, RRID: AB_244268), mouse Ly-6A/E (Sca-1)-PE/Cy5 (BioLegend #108109, RRID: AB_313346), mouse CD117 (c-kit)-PE (BioLegend #105807, RRID: AB_313217), Rat IgG2a, κ Isotype Ctrl-PE/Cy5 (BioLegend #400509), Rat IgG2b, κ Isotype Ctrl-PE (BioLegend #400607; RRID: AB_326551). BrdU labeling of S-phase cells and flow cytometry analysis were done using A488-conjugated anti-BrdU antibodies (BD Biosciences #347580, RRID: AB_400326) as described [50]. For apoptosis assay, Annexin V-APC (MabTag) or CaspGLOW™ Red Active Caspase-3 Staining Kit (Biovision) kits were used according to the manufacturer’s protocols.

### Immunohistochemistry and western blot

Immunoblotting (WB) was performed as described [15]. Cells were lysed in NP-40 lysis buffer (50 mM Tris-HCl, 150 mM NaCl, 5 mM EDTA, 2% NP-40, pH 8.0) supplemented with protease inhibitor (cOmplete ULTRA tablets EASYpack, Roche) and phosphatase inhibitor (PhosSTOP, Roche). For WB the following antibodies were used: anti-p53 (NCL-p53–505, Leica Microsystems, 1:2000, RRID: AB_563932), anti-p21 (F-5, #sc-6246, Santa-Cruz, 1:200, RRID: AB_628073), anti-Cas9 (E7M1H, #19526, Cell Signaling, 1:1000, RRID: AB_2798820), anti-β-actin (AC-15, #ab6276, Abcam, 1:10000, RRID: AB_2223210). Secondary anti-mouse or anti-rabbit IgG-HRP (GE Healthcare, 1:5000) and SuperSignal ECL kit (Thermo Fisher) were used for detection. Tissue samples for histology and immunohistochemistry (IHC) were formalin-fixed and processed as described before [15]. For IHC the following antibodies and kits were used: anti-cleaved caspase-3 (#9661, Cell Signaling, 1:100, RRID: AB_2341188), DeadEnd TM colorimetric TUNEL System (Promega), anti-p53 (NCL-p53–505, Leica Microsystems, 1:1000, RRID: AB_563932), anti-GFP (ab6556, Abcam, 1:500, RRID: AB_305564), anti-BrdU (BU1/75(ICR1), #OBT0030G, Bio-Rad, 1:100, RRID: AB_609567) and biotinylated rabbit anti-mouse IgG (31,834, Invitrogen, 1:500). Detection of senescence-associated β-galactosidase activity (SA-βGal) in frozen tissue sections and in cytospin samples was performed as described [15, 50]. Images were acquired using the Leica Aperio Versa slide scanner and Leica Aperio eSlide Manager software v. 1.0.3.37. Aperio ImageScope software v. 12.3.2.8013 was used for IHC image analysis, quantification was performed using Positive Pixel Count Algorithm v.9 and calculated as the ratio N_positive_/N_total_ pixels in 10 fields of view (1000X1000 pixel each) per sample relative to the mean of the untreated samples as baseline.

### PCR and RT-PCR

PCR detection of the LSL cassette and Cre-mediated recombination in the targeted *Trp53* allele was done as described [15]. For reverse-transcription real-time PCR (RT-qPCR) RNA was isolated from cells or tissue samples using the RNeasy Mini Kit (Qiagen) and cDNA was generated with the SuperScript VILO cDNA Synthesis Kit (Invitrogen). Gene expression was analyzed on a LightCycler 480 (Roche) using SYBR Green (Thermo Fisher Scientific) and primers specific for mouse *Trp53*, *Cdkn1a/p21*, *Ccng1*, *Bbc3/Puma*, *β-Actin/Actb* [50]. Data were evaluated by the ΔΔCt method using β-actin gene expression for normalization.

### Chromatin immunoprecipitation (ChIP)

AML cells were treated and fixed after 48 h with freshly prepared 18.5% (w/v) paraformaldehyde (PFA) for 10 min at RT, aiming at a final concentration of 0.88% (v/v) PFA. Crosslinking of DNA and proteins was terminated by quenching unreacted PFA by addition of glycine to 125 mM end concentration and further incubation for 5 min at RT. Cells were pelleted by centrifugation at 300 x g for 10 min at 4 °C, washed twice with ice-cold PBS and lysed at a concentration of 2 × 10^7^ cells ml^− 1^ in SDS lysis buffer containing protease inhibitor. 250 μl lysate per tube was sonicated to shear DNA to a fragment size of 200–1000 bp, using the Sonicator Bioruptor Twin UCD-400 (Diagenode) for 5 cycles of 30 s ON/ 30 s OFF. After sonication, cell debris was pelleted by centrifugation for 10 min at 10,000 x g at 20 °C. Supernatant containing the sheared chromatin was aliquoted á 100 μl and either frozen at − 80 °C for later use or directly processed. For each antibody, one 100 μl aliquot was used and diluted 1:10 with dilution buffer. Pre-clearing was performed for 1 h at 4 °C using 50 μl blocked Protein G-coupled Sepharose beads (GE Healthcare, 1:1 slurry with 20% EtOH) per sample. Supernatant of pelleted beads (3000 x g, 1 min, 4 °C) was transferred into a new tube and 1% was removed as input DNA and stored at 4 °C. Samples were rotated over night at 4 °C with 2.5 μg antibody: p53 (FL393, Santa Cruz sc-6243) or isotype control (E5Y6Q, Cell Signaling #61656). For precipitation, protein-DNA complexes were bound to blocked beads (50 μl per sample) for 4 h at 4 °C. Beads were washed once with each Low Salt, High Salt and LiCl Immune Complex washing buffers and twice with TE buffer. Crosslinking was reverted by incubating immunoprecipitated samples and inputs at 99 °C for 10 min with 100 μl of 10% (w/v) Chelex 100 solution. Proteins were digested with proteinase K for 30 min at 55 °C and this enzyme was subsequently inactivated at 99 °C for 10 min. DNA was eluted in two steps with ddH_2_O, first with 55 μl and then with 100 μl each time by centrifugation (1 min at 12,000 x g). Analysis of bound DNA fragments were performed using qPCR with 1 μl DNA per reaction using primers specific for p53 response elements in the *Cdkn1a/p21*, *Bax* and *Bbc3/Puma* genes.

### Statistical analysis

For statistical analysis the GraphPad Prism 8 software was used. Graphs show mean values obtained with n technical or biological replicates, and error bars in all figures represent standard deviation (SD), unless indicated otherwise. Two groups were tested for statistically significant differences by a two-sided unpaired t-test or, if not normally distributed, by a Mann-Whitney test. Multiple groups were tested by 1way ANOVA in conjunction with a Dunnett’s multiple comparisons test. *P*-values of 1way ANOVA and selected pairwise comparisons are reported in the respective figures. Three or more groups that have been split on two independent variables (here treatment and genotype) were analyzed by 2way ANOVA in conjunction with Sidak’s multiple comparisons test. *P*-values for the interaction effect between the two independent variables and *P*-values of selected pairwise comparisons are reported in the figures. Survival data were analyzed with pairwise Log-rank (Mantel-Cox) tests. A *p*-value < 0.05 was used as level of significance.

## Results

### Leukemia model for partial p53 reactivation

**Fig. 1:**
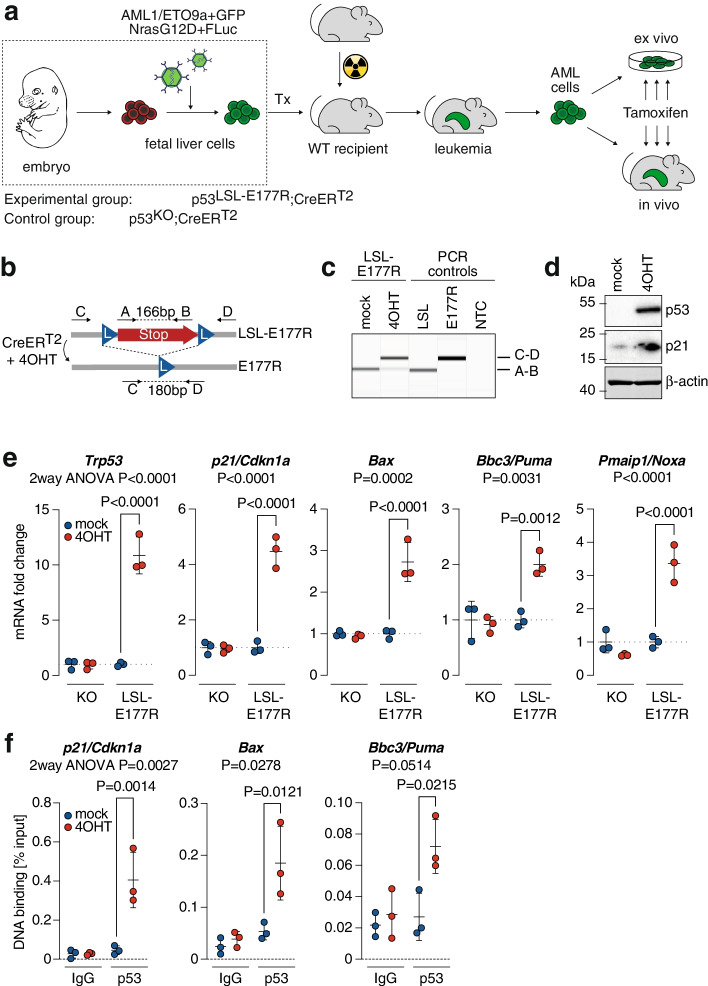
Acute myeloid leukemia model with inducible reactivation of the partial LOF variant p53^E177R^. **a** Experimental outline for in vivo generation of AML by retroviral transduction of fetal liver cells from embryos expressing CreER^T2^ together with constitutive p53 knockout (KO) or Cre-inducible partial LOF p53 variant E177R (LSL-E177R). Leukemia, that developed after transplantation of transduced FLCs into irradiated wild-type recipient mice, was further propagated ex vivo for cell culture studies or transplanted into secondary recipients for in vivo reactivation experiments. **b** Scheme depicting CreER^T2^-mediated excision of the floxed stop cassette (LSL), which silences p53 expression in the LSL-E177R allele. Shown are primers A-D used for genotyping and validating Cre-mediated recombination of the *Trp53* gene locus. **c** PCR genotyping: switch from the A-B to C-D PCR product reveals efficient recombination in *Trp53*^LSL-E177R/LSL-E177R^; *Rosa26*^CreERT2^ AML cells upon 48 h treatment with 1 μM 4OHT. **d** Immunoblot demonstrating induction of p53 and p21/Cdkn1a protein expression upon 4OHT treatment of LSL-E177R; CreER^T2^ (short: LSL) AML cells. β-actin is shown as a loading control. **e** Quantitative reverse transcription PCR for *Trp53* and p53 target gene induction in LSL-E177R; CreER^T2^ and KO; CreER^T2^ AML cells 48 h after treatment with 4OHT. Data were normalized to *Actb* and mock treatment. **f** Chromatin immunoprecipitation analysis of p53 at the indicated target gene promoters 48 h after 4OHT or mock treatment. Chromatin samples were immunoprecipitated with α-p53 antibody (FL393) or IgG as background control. Shown is DNA binding as % input chromatin. **e**-**f** Shown are mean ± SD; datapoints represent biological replicates (*n* = 3); reported are 2way ANOVA *P*-values for the interaction between the two independent variables treatment and genotype and *P*-values for indicated pairwise comparisons (Sidak’s multiple comparisons test)

To model the therapeutic effect of partial p53 reactivation, we have used a previously described genetic Cre-mediated recombination approach to activate a conditional p53 allele in p53-deficient tumor cells [22]. In contrast to earlier studies based on restoration of wild-type p53 [19, 21, 22], we have used a conditional knock-in allele for the p53 partial loss-of-function variant E177R (*Trp53*^LSL-E177R^) in combination with the *Rosa26*^CreERT2^ transgene, which constitutively expresses tamoxifen-regulated CreER^T2^ [15, 22] (Fig. 1a). The inducible CreER^T2^ recombinase can be activated at will by tamoxifen in vivo, or 4-hydroxytamoxifen (4OHT) in vitro, to excise the lox-stop-lox (LSL) cassette that silences expression of the *Trp53*^LSL-E177R^ locus, thus triggering expression of the E177R protein (Fig. 1b). We transduced fetal liver cells from *Trp53*^LSL-E177R;LSL-E177R^;*Rosa26*^CreERT2^ mice with two bicistronic retroviral constructs – one expressing *Nras*^*G12D*^ and firefly luciferase, the second *AML1/ETO9a* and EGFP, and transplanted them into lethally irradiated recipient mice as previously described [46] (Fig. 1a). The mice developed acute myeloid leukemia within 1–2 months. As a control for p53-independent toxicity of tamoxifen and DNA damage induced by Cre, we simultaneously generated CreER^T2^-expressing AML with a constitutive p53 knock-out (KO).

### Partial p53 reactivation inhibits leukemia proliferation and viability

p53-deficient leukemia cells were explanted and further propagated ex vivo. In LSL-E177R;CreER^T2^ (short: LSL) leukemia cells, 4OHT induced excision of the lox-stop-lox (LSL) cassette and resulted in expression of E177R at the mRNA and protein level (Fig. 1c-e). This was accompanied by significantly enhanced expression of p53 target genes such as the cyclin-dependent kinase inhibitor *p21/Cdkn1a.* Of note, we also observed significant induction of several pro-apoptotic target genes including *Bax, Bbc3/Puma* and *Pmaip1/Noxa* (Fig. 1e). As E177R has previously been reported to be unable to bind and transactivate pro-apoptotic target genes [15], we also analyzed binding of E177R to chromatin (Fig. 1f). We not only detected significant levels of E177R binding to the *p21/Cdkn1a* promoter, but also to the *Bax* promoter (Fig. 1f). Moreover, there was also a trend for binding to the *Bbc3/Puma promoter* that reached statistical significance only in a pairwise comparison (Fig. 1f). Notably, no target gene activation was observed in control KO;CreER^T2^ (short: KO) leukemia cells (Fig. 1e).

**Fig. 2:**
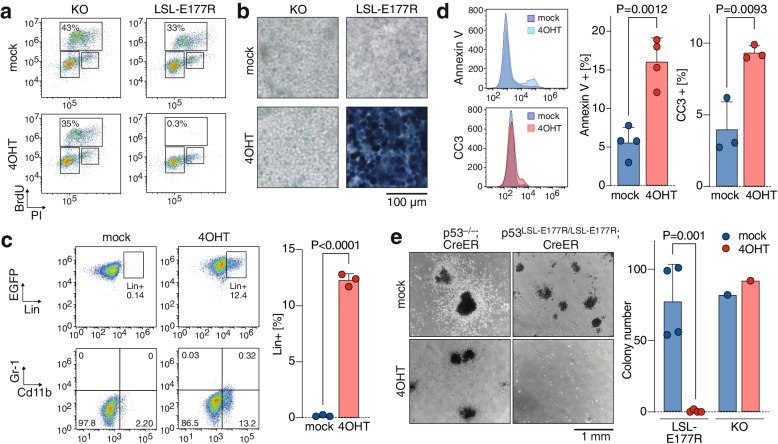
In vitro response of p53-deficient AML to E177R activation. **a** Flow cytometry of BrdU and propidium iodide (PI)-stained LSL-E177R; CreER^T2^ (LSL) AML cells 72 h after indicated treatment. KO; CreER^T2^ (KO) AML cells are shown as controls. The percentage of cells in S phase is indicated. **b** Senescence-associated β-galactosidase staining of LSL and KO AML cells 1 week after indicated treatment. **c** Flow cytometry of LSL AML cells stained for differentiation markers (Lin, CD11b, Gr-1) 48 h after indicated treatment. **d** Flow cytometry of LSL AML cells stained for the apoptosis markers annexin V and cleaved caspase-3 (CC3) 48 h after indicated treatment. **e** Clonogenic growth assay of AML cells with indicated genotype and treatment grown in MethoCult medium for 1 week. In all experiments, cells were treated with 1 μM 4OHT or solvent (ethanol) as the mock control. All quantification bar graphs show mean ± SD; datapoints represent biological replicates; reported are *P*-values of a two-tailed unpaired t-test

Consistent with previous reports demonstrating that E177R is proficient in regulation of cell cycle and senescence [15], activation of E177R inhibited cell cycle progression, resulting in a strongly reduced number of cells in S phase, which was not observed upon tamoxifen treatment of control KO cells (Fig. 2a). At later time points, we observed a massive accumulation of senescent cells marked by expression of senescence-associated beta-galactosidase selectively in tamoxifen-treated LSL but not KO cells (Fig. 2b). Moreover, as induction of differentiation is a recognized tumor-suppressive mechanism exerted by wild-type p53 [21, 54], we tested for signs of differentiation. We observed an increase of the lineage-positive population after 7 days of 4OHT treatment, in particular an enrichment of the population of CD11b-positive cells indicating myeloid differentiation (Fig. 2c). Consistent with robust transactivation of pro-apoptotic target genes (Fig. 1e, f), we also noticed a significant drop in viability of LSL leukemia cells after 4OHT treatment and flow cytometry revealed induction of the apoptosis markers annexin V and cleaved-caspase 3 after expression of E177R (Fig. 2d). Importantly, activation of E177R abolished the clonogenicity of leukemia cells in 3D culture, whereas KO cells remained unaffected excluding p53-independent toxicity of 4OHT or Cre as a cause (Fig. 2e). Together, these experiments demonstrate that genetic reactivation of E177R induces multiple tumor-suppressive programs in AML cells that induce cell cycle withdrawal or cell death and prevent clonogenic expansion.

### Partial p53 reactivation induces leukemia regression

**Fig. 3:**
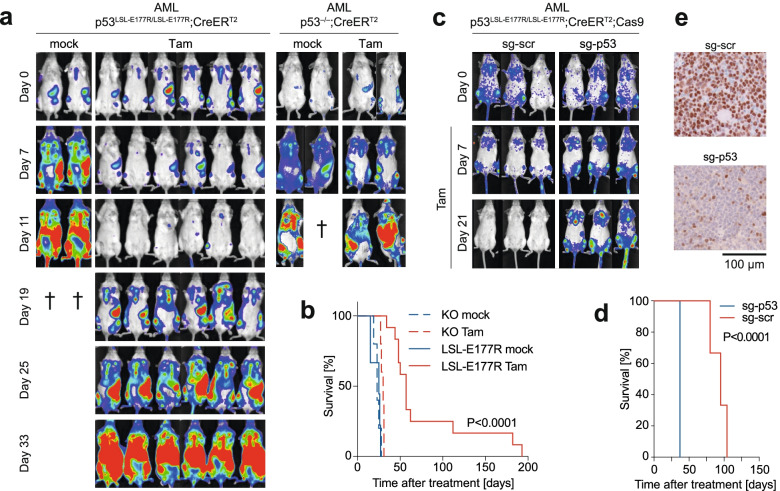
E177R activation induces regression of p53-deficient AML in vivo. **a**-**b** 10^6^ AML cells of indicated genotype were transplanted into sublethally irradiated 129X1 albino x B6 albino (F1 hybrid) mice. After engraftment was confirmed by bioluminescence imaging (BLI), mice were treated 1 week with daily i.p. injections of either vehicle (corn oil) or 1 mg tamoxifen. Mice were euthanized when humane endpoint criteria were reached. **a** BLI of representative mice. **b** Kaplan-Meier survival graph. Log-rank (Mantel-Cox) test for LSL mock vs. Tam. **c**-**d** 10^6^ LSL-Cas9 AML cells transduced ex vivo with lentivirus encoding either *Trp53*-directed sgRNA (sg-p53) or scrambled control sgRNA (sg-scr) were transplanted into mice as in **a** and treated 1 week with daily i.p. injections of 1 mg tamoxifen. Mice were euthanized when humane endpoint criteria were reached. **c** BLI of both cohorts. **d** Kaplan-Meier survival graph. Log-rank (Mantel-Cox) test. **e** Immunohistochemistry for p53 of relapsed leukemia samples from both cohorts

Encouraged by the in vitro data, we transplanted LSL and KO AML cells (both containing CreER^T2^) into sublethally irradiated recipient mice and monitored transplant engraftment, leukemia progression and therapy response by bioluminescence imaging (BLI) of the firefly luciferase co-expressed with *Nras*^G12D^ (Fig. 3 a). After detection of bioluminescence in tubular bones, sternum and spleen as a sign of successful leukemia engraftment, each cohort was randomly subdivided into two groups which were injected for 1 week daily with either tamoxifen (Tam) or solvent (mock) as control. As revealed by BLI and monitoring of clinical symptoms, leukemia quickly progressed in both mock-treated genotypes (median survival for LSL 25 days, for KO 23 days) (Fig. 3b). When the LSL group was treated with tamoxifen a strong clinical response was observed: BLI showed a gradual decrease of luciferase signal to almost background levels within 2 weeks of treatment (Fig. 3a). Median survival was extended more than twice in comparison to the control group and reached 57 days, 3/12 mice survived for > 100 days and 2 for almost 200 days (Fig. 3b). Importantly, tamoxifen administration had only minor effect on survival of mice with KO leukemia, which continued to progress under treatment (Fig. 3a), and provided only a subtle survival advantage (median survival 30 days, Fig. 3b), underscoring the role of E177R as the driver of the therapeutic response.

To confirm that the therapy response is indeed dependent on E177R expression, we generated an isogenic LSL-E177R;CreER^T2^ AML cell clone with stable Cas9 expression and subsequently transduced this clone with lentiviruses expressing either a *Trp53*-targeting (sg-p53) or a non-targeting scrambled sgRNA (sg-scr). Both sg-p53 and sg-scr AML cell types were transplanted into sublethally irradiated recipient mice and, following successful engraftment, treated with tamoxifen as described above. As indicated by BLI and survival analysis, sg-p53 AML did not respond to tamoxifen, whereas the same treatment promoted regression of sg-scr leukemia and resulted in a strong survival benefit (median survival 37 and 95 days, respectively) (Fig. 3c and d). Immunohistochemistry confirmed the absence of p53 protein in sg-p53 AML after tamoxifen administration, whereas sg-scr leukemia samples collected after disease relapse were strongly positive for p53 protein (Fig. 3e). Together, these results indicate that E177R is mediating the reactivation response and strongly suggest that even a partial restoration of p53 activity can provide a significant therapeutic effect in vivo.

**Fig. 4:**
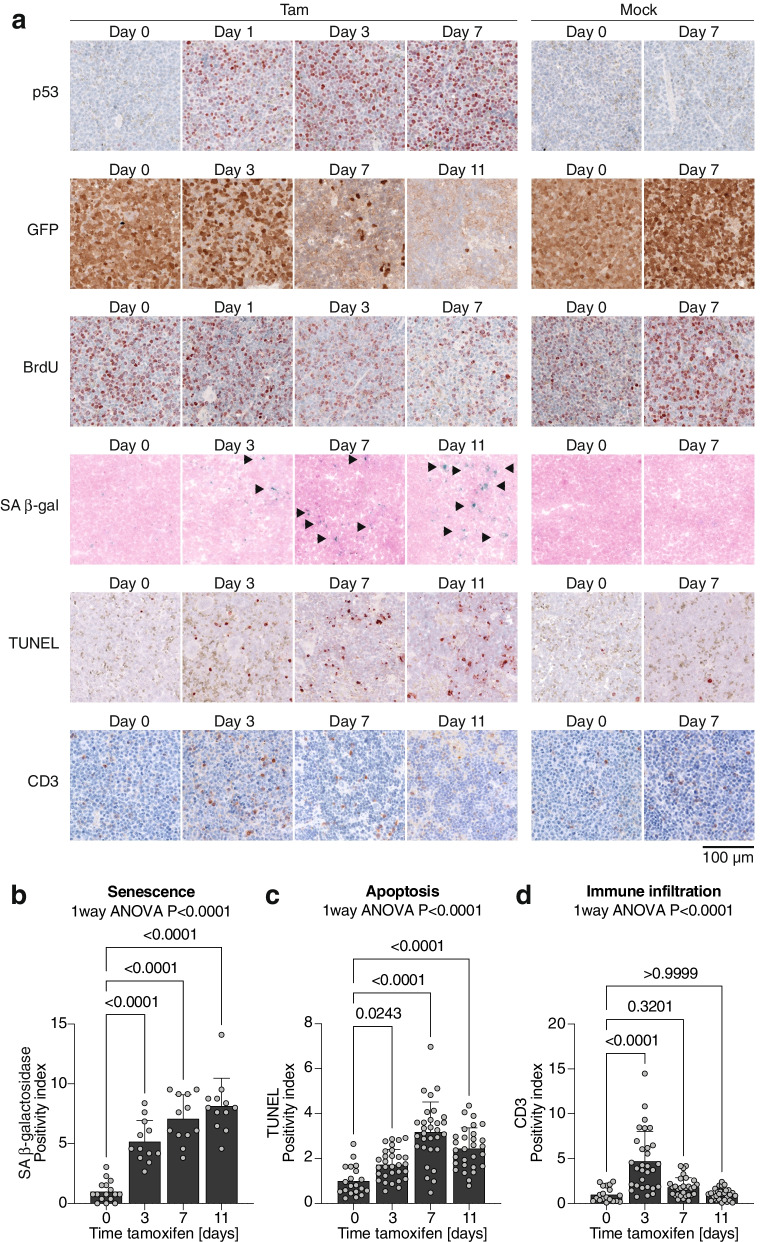
In vivo response of p53-deficient AML to E177R activation. **a** Mice transplanted with LSL AML cells were sacrificed before and day 1, 3, 7 and 11 after start of 1 week of daily treatment with Tam or corn oil (mock). Samples were analyzed by immunohistochemistry for p53^E177R^, GFP (as a marker of AML cells), BrdU (proliferation), senescence-associated β-galactosidase (SA β-gal), apoptosis (TUNEL) and CD3 as a marker of immune infiltration. Arrowheads indicate senescent cells. **b**-**d** Quantification of immunohistochemistry analysis. **b** Senescence-associated β-galactosidase. **c** Apoptosis (TUNEL). **d** Immune infiltrate (CD3). All bar graphs show the mean positivity index ±SD; datapoints represent individual fields of view (1000X1000 pixel each) from *n* = 2 mice sacrificed at each time point; reported are *P*-values of 1way ANOVA and indicated pairwise comparisons (Dunnett’s multiple comparisons test)

The remarkable efficiency of reactivation therapy prompted us to investigate the mechanisms that underlie the E177R-mediated response. Using immunohistochemistry, we analyzed tissue samples collected at different time-points after therapy start. As expected, we observed fast accumulation of p53 protein in LSL- tumors after the start of daily tamoxifen injections (Fig. 4a). Interestingly, all relapsed tumors retained a high expression level of E177R protein (Fig. 3e), indicating that relapse is not driven by incomplete Cre-mediated E177R activation or secondary loss of E177R expression. It rather suggests that some leukemia cells tolerate the residual tumor suppressive activity of E177R and eventually adapt to its presence. Coherent with the loss of the luciferase signal in BLI, E177R induction led to a gradual elimination of GFP-positive leukemia cells (Fig. 4a). Consistent with E177R being proficient in cell cycle inhibition and senescence induction, we detected a marked drop in proliferation in tamoxifen-treated leukemia, as indicated by reduced number of BrdU-positive cells (Fig. 4a). In parallel, we observed a significant accumulation of senescent cells (Fig. 4a and b). However, while cell cycle arrest and senescence can decelerate or even stop tumor growth, these processes did not fully explain the rapid removal of leukemia cells, which became detectable as early as 3 days and was clearly evident 7 days after the first tamoxifen administration (Fig. 4a). As we had observed induction of apoptosis following E177R induction in vitro, we therefore also analyzed apoptosis levels immunohistochemically in tissue samples by TUNEL assay and observed a significant increase in tamoxifen-treated, but not in mock-treated, tumors (Fig. 4a and c). Thus, similar to the in vitro results, in vivo induction of E177R also triggered a broad spectrum of tumor suppressive programs, including cell cycle arrest, senescence and apoptosis.

Restoration of wild-type p53 in liver cancer was shown to trigger an infiltration by multiple components of the immune system which contributed to clearance of senescent tumor cells and accelerated cancer regression [21]. Using CD3 as a surrogate marker, we observed significantly increased staining at day 3 in tamoxifen-, but not mock-treated tumors (Fig. 4a and d), suggesting that immune cells might contribute to E177R-mediated leukemia regression.

### Partial p53 reactivation induces regression of Myc-driven Burkitt-like lymphoma

**Fig. 5:**
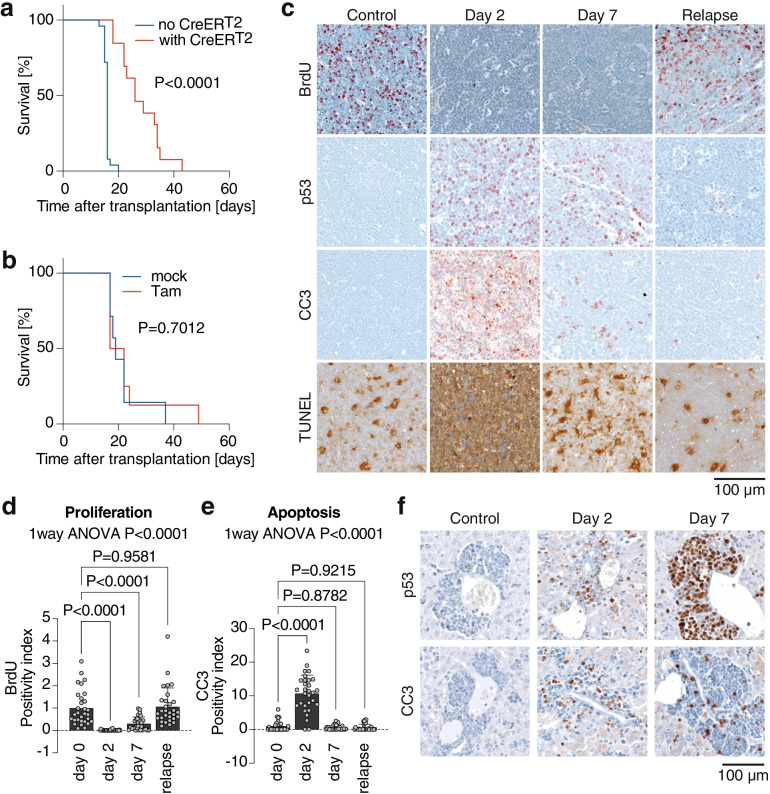
E177R activation induces regression of p53-deficient Burkitt-like B cell lymphomas. **a** EμMyc; *Trp53*^LSL-E177R/−^ lymphomas with (*n* = 13) or without (*n* = 25) the *Rosa26*^CreERT2^ transgene were transplanted into immunocompetent 129/B6 F1 hybrid mice and, after confirmation of disease onset, treated 1 week with daily i.p. injections of 1 mg tamoxifen. Mice were euthanized when humane endpoint criteria were reached. Shown is the Kaplan-Meier survival graph with Log-rank (Mantel-Cox) test. **b** EμMyc; *Rosa26*^CreERT2/+^ lymphomas were transplanted as in **a** and treated 1 week with daily i.p. injections of either corn oil (mock; *n* = 7) or 1 mg tamoxifen (Tam; *n* = 8). Shown is the Kaplan-Meier survival graph with Log-rank (Mantel-Cox) test. **c**-**f** Mice transplanted with EμMyc; *Trp53*^LSL-E177R/−^; *Rosa26*^CreERT2^ lymphomas were sacrificed before, day 2 and 7 after start of tamoxifen (Tam) treatment or upon relapse. Samples were analyzed by immunohistochemistry for BrdU (proliferation), p53 and apoptosis (cleaved caspase-3 CC3, TUNEL). **c** Representative images. **d**-**e** Quantification of immunohistochemistry. **d** Proliferation (BrdU). **e** Apoptosis (cleaved caspase-3, CC3). All bar graphs show the mean positivity index ±SD; datapoints represent individual fields of view (1000X1000 pixel each) from *n* = 3 mice sacrificed at each time point; reported are *P*-values of 1way ANOVA and indicated pairwise comparisons (Dunnett’s multiple comparisons test). **f** Representative images of hepatic lymphoma infiltrates in the periportal space stained for p53 and apoptosis (cleaved caspase-3, CC3) at indicated time points after Tam treatment

Inspired by the results in the AML model, we decided to test whether partial restoration of p53 function is an effective treatment in other hematological malignancies such as lymphoma. We first studied EμMyc-driven B cell lymphoma, which mimics the t (8;14)(q24;q32) translocation in human Burkitt lymphoma and which is facilitated by the E177R mutation [15, 48]. We crossed male *EμMyc* mice with female *Trp53*^LSL-E177R/+^;*Rosa26*^CreERT2^ mice to generate littermates that undergo LOH and develop p53-deficient LSL-E177R lymphomas with and without expression of CreER^T2^. These p53-deficient lymphoma cells were transplanted into immunocompetent recipient mice. After successful engraftment of the lymphoma, detected by palpable lymph nodes, all animals were treated with tamoxifen for 7 consecutive days (Fig. 5a). In the group of mice with CreER^T2^, tamoxifen treatment activated E177R and triggered a pronounced therapeutic response, as suggested by a significantly extended survival compared to tamoxifen-treated mice with LSL-lymphomas lacking CreER^T2^ (median survival 26 vs. 16 days, Log-rank (Mantel-Cox) test *P* < 0.0001; Fig. 5a). Excluding a confounding effect of CreER^T2^, no difference in survival was observed in mice lacking an inducible p53 allele (Fig. 5b). Although tamoxifen also functions as an anti-estrogen, we observed no gender-dependent differences in the p53 reactivation response (Supplemental Fig. S1).

Expression of E177R protein was readily detected by immunohistochemistry in tumors 2 days after the therapy start accompanied by strong inhibition of cell proliferation, evident from the massive reduction in BrdU-positive cells (Fig. 5c and d). Similar as in the AML model, we again detected a massive increase in apoptosis, which culminated at 2 days after therapy start and then decreased to background levels (Fig. 5c and e). Notably, p53-deficient lymphomas are highly invasive and infiltrate into various nonlymphoid organs as evidenced by periportal invasion and spreading of lymphoma cell clusters throughout the liver parenchyma [55]. Interestingly, we observed robust E177R expression and apoptosis induction also in periportal lymphoma infiltrates (Fig. 5f), suggesting that partial reactivation is equally effective in a metastatic setting.

At later time-points, the number of p53-positive cells dropped and relapsed tumors were mostly negative for p53 staining with only a few remaining small clusters of p53-positive cells (Fig. 5c). This suggests that recombination in this model is either incomplete or recombined LSL-E177R becomes inactivated by, for example, LOH, so that residual or emerging p53-deficient lymphoma cells escape and mediate relapse.

### Partial p53 reactivation induces regression of spontaneous T-cell lymphomas in p53-null mice

**Fig. 6:**
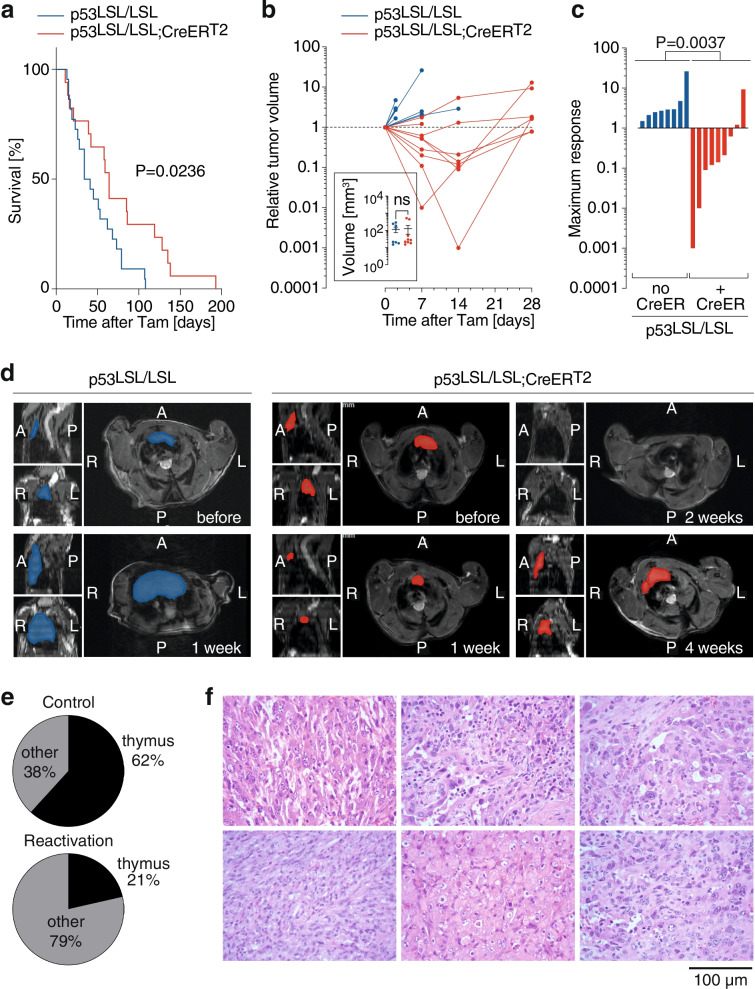
E177R activation induces regression of thymic lymphomas in p53-deficient mice. *Trp53*^LSL-E177R/LSL-E177R^ mice (p53^LSL/LSL^) with the *Rosa26*^CreERT2^ (CreER^T2^) transgene (*n* = 17) were treated 1 week with daily i.p. injections of 1 mg tamoxifen at 4 months of age and euthanized when humane endpoint criteria were reached. p53^LSL/LSL^ mice (without CreER^T2^) treated with tamoxifen served as controls (*n* = 22). **a** Kaplan-Meier survival graph with Log-rank (Mantel-Cox) test. **b**-**d** Individual mice (p53^LSL/LSL^; CreER^T2^ (red): *n* = 9; p53^LSL/LSL^ (blue): *n* = 8) that were diagnosed with thymic lymphoma by MRI received tamoxifen treatment and were re-examined by MRI 7, 14, and 28 days after treatment start. **b** Shown is the fold-change in tumor volume (determined by MRI) relative to pre-treatment for individual animals. Insert shows the mean absolute tumor volume ± SD in both cohorts at the start of treatment; data points represent individual mice; two-tailed unpaired t-test. **c** Shown is the maximum response (fold increase in tumor volume) in individual mice. Reported is the *P*-value of a two-tailed Mann-Whitney test comparing tamoxifen treatment responses of p53^LSL/LSL^; CreER^T2^ (red) and p53^LSL/LSL^ (blue) mice. **d** Representative MRI results from one mouse of each cohort. The p53^LSL/LSL^ mouse without CreER^T2^ (blue) progressed under treatment and reached the endpoint before day 14. The p53^LSL/LSL^ mouse with CreER^T2^ (red) showed tumor regression until day 14 and presented with relapse on day 28. Shown are tumor sections in all 3 dimensions; tumors are highlighted in color. **e**-**f** Cancer types detected in both cohorts after treatment when the animals reached the humane endpoint criteria. **e** Fraction of thymic lymphomas versus non-thymic tumor types (including mostly sarcomas and splenic B cell lymphomas). **f** Representative histological images of non-regressed undifferentiated sarcomas in *n* = 6 different animals

As a second lymphoma model, we decided to use thymic T-cell lymphoma that accounts for the majority of all cancers detected in p53-null mice and spontaneously develops early within the first 6 months of age [49, 56]. We generated *Trp53*^LSL-E177R/LSL-E177R^ mice which co-expressed the *Rosa26*^CreERT2^ transgene (short: p53^LSL/LSL^;CreER^T2^). We used Cre-negative mice that cannot induce E177R expression upon tamoxifen administration as a constitutive p53-null control group (short: p53^LSL/LSL^). In our previous experiments, we estimated the median survival of p53-deficient mice to 5–6 months and therefore expected that the majority of animals should have developed tumors by 4 months of age. As soon as animals reached 4 months of age, both groups were treated 1 week daily with tamoxifen and monitored for survival. This approach allowed us to avoid a potential bias caused by p53-independent effects of tamoxifen. If re-expression of E177R can block or slow down progression of such tumors, it should result in enhanced survival. Indeed, we observed a significantly longer median survival in the E177R reactivation group (64 vs. 37 days survival after therapy end, Fig. 6a).

From this experiment, however, we could not conclude whether re-expression of E177R prevented tumor formation, limited cancer progression or induced regression of established tumors. To address this question, we examined p53^LSL/LSL^ animals with and without CreER^T2^ by MRI. Included were 4-months old or younger mice with clinical signs of thymoma (breath shortening, hunchback posture, weight loss). Animals with thymus enlargement were treated with tamoxifen as described above and monitored by MRI for up to 4 weeks. At the start of therapy both cohorts had a similar average tumor volume (Fig. 6b). In conformity with survival data, imaging showed that all p53^LSL/LSL^ animals without Cre showed progressive tumor growth under tamoxifen (Fig. 6b-d). In stark contrast, we observed a clear reduction of thymus size in 7 of 9 p53^LSL/LSL^ mice with Cre 1–2 weeks after E177R activation (Fig. 6b-d). The response to tamoxifen was similar in mice of both sexes (Supplemental Fig. S2). Moreover, all control animals reached the humane endpoint and had to be sacrificed within 2 weeks after therapy start whereas 7/9 mice with E177R activation survived for more than 4 weeks. Thus, E177R expression elicited in B- and T-cell lymphoma models a similar therapeutic response as in AML, indicating that even partial restoration of p53 function is able to induce regression in different hematopoietic cancers.

Of note, after initial remission thymic lymphomas regrew in virtually all mice. Nevertheless, many of these animals did not die of thymic lymphoma but were sacrificed because of other cancers, mostly sarcomas in varying locations and some splenic B cell lymphomas (Fig. 6e and f). These were not detected as the major tumor before treatment, but apparently continued to grow despite E177R activation, suggesting that not all types of cancer might be equally vulnerable to partial p53 activation.

## Discussion

Previous studies have indicated that many p53-deficient tumors regress when p53 is restored, implying that they are strongly addicted to the absence of p53 activity [19–22, 24]. However, it has remained unclear how much p53 activity is required to trigger tumor regression [18]. Given the yet limited pharmacological abilities to fully reactivate a p53 mutant [25, 26], we aimed to analyze whether partial p53 reactivation may suffice to induce a therapeutic effect. To reproducibly reactivate p53 to a defined suboptimal degree, we chose to genetically limit p53 activity using the previously well-characterized partial LOF variant E177R [15]. This mutation does not affect any DNA contacting residues and maintains a normally folded DNA binding domain [41]. Nevertheless, it is impaired in DNA binding because it fails to form a strong intermolecular salt-bridge which is crucial to stabilize the tetrameric protein complex on DNA [43]. Previous DNA binding studies in vitro and in vivo have characterized the DNA binding defect as a global reduction in DNA binding across the entire target gene spectrum [44, 45]. By limiting global DNA binding in a genetically fixed manner, the E177R mutation is suitable to model the consequences of an incompletely reactivated p53 mutant.

Even though the E177R mutation reduced DNA binding globally, we observed transactivation of the typical p53 target genes (Fig. 1e) and induction of a broad range of effector programs including cell cycle arrest, senescence, differentiation and apoptosis (Fig. 2). While transactivation of antiproliferative target genes like *p21/Cdkn1a* is in line with the described phenotype of E177R knock-in mice and explains induction of cell cycle arrest, senescence and differentiation [15], activation pro-apoptotic target genes was rather unexpected, because E177R and several other related cooperativity mutants are generally characterized by an apoptosis defect [43, 57–59]. However, the ability of p53 to induce apoptosis is highly context-dependent and modulated by the extent and dynamics of p53 protein accumulation as well as the intrinsic apoptosis threshold of the cell and its level of mitochondrial priming [60–64]. It is therefore conceivable that sustained high-level expression of a DNA-binding impaired p53 mutant can trigger apoptosis especially in cell types with an intrinsically low apoptosis threshold [65, 66]. In line, massive constitutive stabilization of the E177R mutant protein by knockout of Mdm2 was shown to trigger lethal apoptosis in highly proliferative embryonic tissues [65], which have a lower apoptosis threshold than most adult tissues due to Myc-mediated mitochondrial priming [65, 66]. Similarly, tumors in E177R mice commonly display constitutive stabilization of the E177R mutant protein, and DNA damage in such tumors was reported to induce pro-apoptotic p53 target genes and render them sensitive to chemotherapy [47]. In all our leukemia and lymphoma models, the E177R protein was readily detectable by immunohistochemistry (Figs. 3e, 4a, 5f), showing a staining pattern similar to human tumors with a massively stabilized mutant p53 protein. Moreover, p53-deficient leukemias and lymphomas commonly upregulate p19Arf [20, 47], which can sequester Mdm2, thereby contribute to the massive E177R accumulation, overcome the apoptosis-deficiency and enable tumor cell killing by E177R.

In vivo, additional non-cell-autonomous p53 effects might contribute to eradication of tumor cells [21, 67, 68]. In liver carcinomas, for example, wild-type p53 restoration triggers infiltration by innate immune cells like macrophages, neutrophils and lymphocytes that support clearance of senescent tumor cells [21]. While we have not detected changes in macrophages, we have observed lymphocytic infiltration upon E177R activation (Fig. 4), which makes it tempting to speculate that immune infiltration induced by p53 in a non-cell-autonomous manner might contribute to tumor regression in vivo. In summary, the presented data indicate that E177R is directly capable of inducing apoptosis in p53-deficient leukemia and lymphoma cells and provide an explanation for the observed cancer regression. Whether and how immune cells contribute remains to be investigated.

We observed cancer regression not only in AML but also in lymphoma models, including thymic T cell lymphomas that spontaneously develop in p53-deficient mice. While non-reactivated mice mostly succumbed to thymic lymphoma, reactivated mice at the time of sacrifice more often presented with other types of cancer, often sarcomas (Fig. 6e and f), suggesting that lymphomas are more vulnerable to p53 reactivation than other cancer entities. A differential sensitivity of sarcomas and lymphomas was also reported upon restoration of wild-type p53, where restoration in lymphomas caused widespread apoptosis, whereas sarcomas showed a delayed anti-proliferative response with features of senescence [22]. The higher sensitivity of hematopoietic cancers is not entirely unexpected as already the normal bone marrow displays an exquisite vulnerability to elevated p53 activity which is at least partially explained by mitochondrial priming of the hematopoietic compartment [61, 66, 69–72]. Moreover, this is in line with clinical studies on p53/mutp53-reactivating compounds like Mdm2-inhibitors or eprenetapopt (APR-246), which have reported clinical responses mostly in patients with hematological cancers [37, 38, 73].

Even though most animals demonstrated strong responses to partial reactivation, none of the animals was cured and all relapsed eventually. The cause of relapse differed between the different models. Relapsed EμMyc-driven lymphomas showed a high percentage of p53-negative tumor cells (Fig. 5c), suggesting that not all lymphoma cells had recombined the LSL-E177R allele and therefore escaped due to a technically inefficient reactivation. An alternative explanation would be a secondary loss or inactivation of the E177R mutant, for example, by LOH. In contrast, relapsed AML mice showed homogeneous high-level expression of the E177R mutant, indicating that some tumor cells eventually adapt and tolerate E177R expression. Of note, these observations are not unique to partial p53 reactivation and similar findings have been reported in the previous studies with wild-type p53 restoration. All EμMyc lymphomas with tamoxifen-inducible p53ER^TAM^ activity relapsed after reactivation, losing either p53ER^TAM^ expression or deleting its upstream activator p19ARF [19]. Responses to partial reactivation are therefore mostly transient, calling for synergistic approaches such as DNA damaging chemotherapeutics that might help to boost p53 activity to obtain longer-lasting remissions. In AML, even a transient response might be efficient, by inducing the clinical remission needed for a bone marrow transplant as the final curative treatment.

Tamoxifen, used to induce E177R-mediated tumor regression in our study, is an estrogen receptor (ER) antagonist. As estrogens vary between sexes and interfere with the p53 pathway [74], the anti-estrogenic tamoxifen activity might have contributed to the p53 reactivation response in a sex-dependent manner. In the AML model, both the LSL and KO leukemia cells were derived from female embryos and 86% of all recipient mice were also female. The improvement in survival upon tamoxifen treatment of LSL-E177R AML mice remained highly significant when excluding male mice from the survival analysis (Supplemental Fig. S3). A gender-effect as the cause for the differential in vitro and in vivo response to tamoxifen treatment can therefore be excluded. However, due to the small number of male recipient mice, we could not analyze whether the reactivation response is different in male AML mice. Both lymphoma models comprised comparable numbers of male and female mice and the reactivation responses were similar in both sexes (Supplemental Fig. S1 and S2). In the transplanted Myc lymphomas, reactivation was also independent of the sex of the lymphoma donor (Supplemental Fig. S1). Together, these analyses indicate no confounding sex-dependent effects in the hematopoietic cancer models studied here. This is in line with the clinical use of tamoxifen primarily for the treatment of hormone-dependent ER+ breast and endometrial cancer. In other ER-negative cancer types, anti-tumor effects of tamoxifen require doses 4–8 fold higher than necessary for ER inhibition [75], suggesting that these effects are hormone-independent.

Of note, tumor cells can not only become addicted to the loss of wild-type p53 activity, they can also become dependent on neomorphic GOF properties of the p53 mutant. Refolding a GOF mutant would therefore not only restore some degree of wild-type function but simultaneously deprive tumor cells of survival-promoting GOF effects. As such, it has been demonstrated that loss of GOF activities by therapeutic ablation of the mutant protein can be sufficient to induce cancer regression, even in the absence of any restoration of wild-type function [76, 77]. By switching p53-null tumor cells to E177R, we can exclude that the responses observed in our cancer models are mediated by a loss of GOF properties. However, it is reasonable to assume that an additional loss of GOF would synergize with restoration of wild-type function and could further enhance the extent and duration of tumor regression. It is therefore expected that a partial p53 reactivation would be even more effective in tumors bearing GOF missense mutants rather than pure LOF mutants.

## Conclusions

We demonstrate that a p53 variant, which has partially lost the activity of wild-type p53 and by itself increases cancer susceptibility and promotes oncogene-driven development of leukemia and lymphoma, induces cancer regression in vivo when introduced into p53-deficient leukemia and lymphomas. This not only provides compelling evidence that p53 mutants function in a highly context-dependent manner, being pro-tumorigenic in one setting and tumor suppressive in another, it also highlights how sensitive cancer cells respond to changes in p53 activity and that changes in activity are more important than the absolute activity level. Even a slight increase in p53 activity resulting from switching a p53-null into a partially active p53 allele can suffice to induce tumor regression. As such, our findings will encourage ongoing research into mutant p53-reactivating drugs by providing genetic proof-of-principle evidence that incomplete reactivation of p53 mutants can elicit a beneficial therapeutic response.

## Supplementary Information

**Additional file 1.**

## Data Availability

All data generated or analyzed during the present study are included in this published article.

## Abbreviations

LOF: Loss of function
GOF: Gain of function
MDS: Myelodysplastic syndrome
AML: Acute myeloid leukemia
GSH: Glutathione
Tam: Tamoxifen
4OHT: 4-hydroxy-tamoxifen
LSL: loxP-stop-loxP
MRI: Magnetic resonance imaging
BLI: Bioluminescence imaging
LOH: Loss of heterozygosity
BrdU: Bromodeoxyuridine
GFP: Green fluorescent protein
RT: Room temperature
PBS: Phosphate buffered saline
