# Supplementary material for: Partial p53 reactivation is sufficient to induce cancer regression
Source: J Exp Clin Cancer Res. 2022 Mar 2;41:80. doi: 10.1186/s13046-022-02269-6 (PMC8889716; doi:10.1186/s13046-022-02269-6)
Supplement: Supplementary file 1 — Additional file 1. [file 13046_2022_2269_MOESM1_ESM.pdf]

Supplemental Information

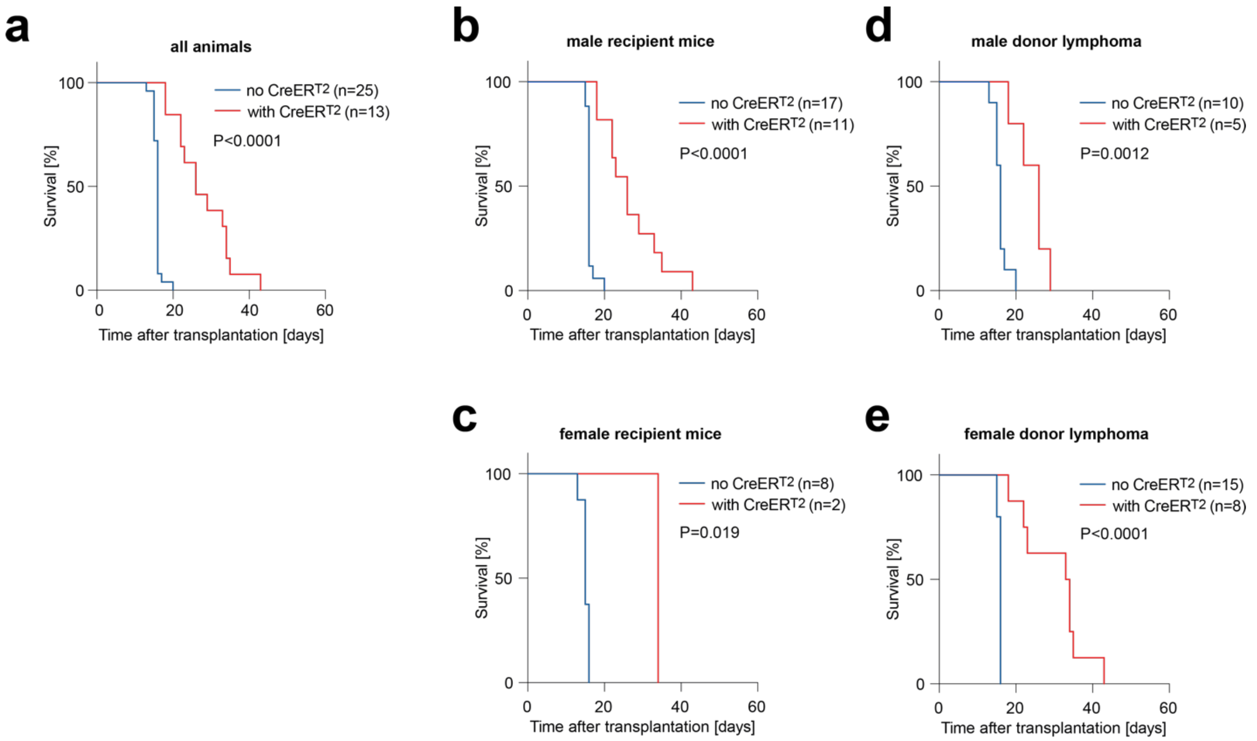

Supplemental Fig. S1. EμMyc; *Trp53*$^{\text{LSL-E177R/-}}$ lymphomas with (n=13) or without (n=25) the *Rosa26*$^{\text{CreERT2}}$ transgene were transplanted into immunocompetent 129/B6 F1 hybrid mice and, after confirmation of disease onset, treated 1 week with daily i.p. injections of 1 mg tamoxifen. Mice were euthanized when humane endpoint criteria were reached. Shown are Kaplan-Meier survival graphs with P-values of the Log-rank (Mantel-Cox) test. **a** all animals in the study. **b** only male recipient mice. **c** only female recipient mice. **d** only animals transplanted with lymphoma cells from male donor mice. **e** only animals transplanted with lymphoma cells from female donor mice.

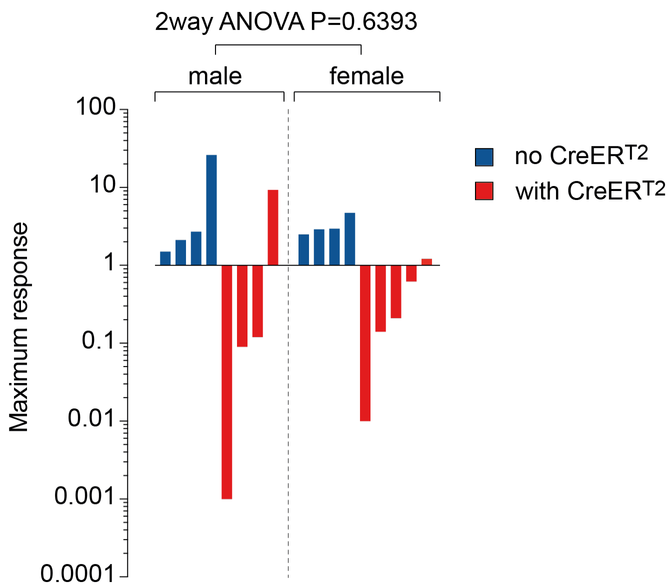

Supplemental Fig. S2 *Trp53*$^{LSL-E177R/LSL-E177R}$ mice (p53$^{LSL/LSL}$) with or without the *Rosa26*^{CreERT2} (CreER^{T2}) transgene that were diagnosed with thymic lymphoma by MRI were treated 1 week with daily i.p. injections of 1 mg tamoxifen and were re-examined by MRI 7, 14, and 28 days after treatment start. Shown is the maximum response (fold increase in tumor volume) in individual male and female mice. Reported is the P-value of of a 2way ANOVA analysis for the interaction between sex and genotype/reactivation.

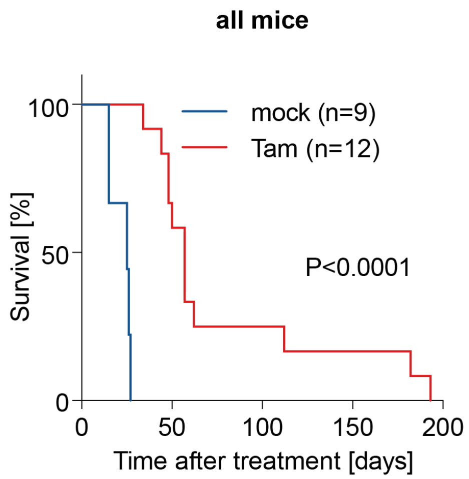

Supplemental Fig. S3 Female LSL-E177R; CreER^{T2} (LSL) AML cells were transplanted into sublethally irradiated 129X1 albino x B6 albino (F1 hybrid) mice. After engraftment was confirmed by bioluminescence imaging (BLI), mice were treated 1 week with daily i.p. injections of either vehicle (corn oil) or 1 mg tamoxifen. Mice were euthanized when humane endpoint criteria were reached. Shown are Kaplan-Meier survival graphs for all (left) and female-only recipient mice (right). Reported are P-values of the Log-rank (Mantel-Cox) test for mock vs. Tam.
